# Notes on Subinvolution

**Published:** 1879-04

**Authors:** D. T. Nelson

**Affiliations:** Professor of Physiology, Chicago Medical College


### Original Communications.

ARTICLE II.

NOTES ON SUBINVOLUTION. BY D. T. NELSON, M.D., Professor of Physiology, Chicago Medical College. Read before the Gynæcological Society, February 28, 1879.

As early as 1843 Sir James Simpson called the attention of the profession to this condition of the uterus, under the name of "Morbid Permanence of the State of Puerperal Hypertrophy," and in the same paper described his uterine sound—now so well and favorably known—as an efficient instrument in the diagnosis of this and many other diseases in and around the uterus.

In another paper, read before the Edinburgh Obstetric Society in 1852, he treats of the same subject under the title "Morbid Deficiency and Morbid Excess in the Involution of the Uterus After Delivery." In this paper he uses the term super-involution to designate an excess, and the word subinvolution to indicate a deficiency in the normal involution after delivery.

Since this last paper by Prof. Simpson, subinvolution has been recognized as a form of uterine diseases, but it was to insist upon the importance of its early recognition and appropriate treatment that I proposed this as the topic for discussion this evening.

Prof. Simpson, in the paper referred to, so graphically describes the growth of the pregnant uterus and its involution after delivery, that I cannot do better than read his description. "It is," he says, "a kind of physiological hypertrophy, unequalled, either in regard to its magnitude or its rapidity, in any other organ in the adult human body; for, during the forty weeks of utero-gestation, the uterus enlarges from nearly 3 inches in length and $1\frac{3}{4}$ inches in breadth to 12 or 15 inches in length and 9 to 10 inches in breadth. It increases from about 2 ounces in weight to 25 or 30 ounces. The cavity of the uterus before impregnation is less than one cubic inch, while at the full term of pregnancy it is extended to above 400 cubic inches; and the surface of the organ increases from about 5 or 6 square inches to nearly 350 square inches. Before impregnation, the uterine cavity would not hold above a dram or two of fluid; at the end of the ninth month of utero-gestation, its contents usually weigh from 120 to 150 ounces.

The rapidity, however, with which the uterus diminishes in size after delivery is perhaps still more marvellous than the rapidity with which it increases in size after impregnation; for, while it takes forty weeks to attain the dimensions of the fully developed uterus of pregnancy, it requires only from four to eight weeks to decrease to the small size of the same organ in its impregnated condition."

While such is the normal course during involution of the uterus, there are, unfortunately, very many cases in which it does not pursue this natural course, but, for a variety of reasons, after miscarriage especially, and after labor at full term as well, it remains permanently enlarged.

What then are the causes of subinvolution?

In regard to time, they may be arranged in three groups.

1. Those operating previous to the pregnancy.
2. Those occurring at the date of confinement.
3. Those subsequent to delivery.

1. Those operating previous to the pregnancy.

*a.* Constitutional diseases impairing muscular strength and nervous power, such as tuberculosis, scrofula, syphilis and the like.

*b.* Local disease, interfering with the normal functions of the pelvic organs, such as pelvic peritonitis and cellulitis, with their subsequent adhesions.

*c.* Tumors in or near the uterus, such as fibroid and fibrocystic with extra-uterine fœtation.

*d.* Enlargement of the uterus, the result of previous miscarriage or pregnant labors.

2. Those occurring at the time of confinement.

*a.* Over-distention of the uterine walls; by a large fœtus, unusual quantity of liquor amnii, twins and the like.

*b.* Delay in delivery from the numerous causes of tedious labor.

*c.* Parturient injuries of the uterus and its appendages, such as rupture of the walls of the womb, laceration of the cervix, vagina and perineum; injuries of the uterus, or its appendages, in the production of a miscarriage; adherent placenta; flooding, producing clots to be retained.

3. Those occurring subsequent to delivery.

*a.* Puerperal fever, in its various forms of septicæmia, peritonitis, metritis and, possibly, something more than a combination of these in the epidemic form.

*b.* And then the milder types of fever, such as intermittent fever—the fever attending the disorders of the breasts; phlegmasia alba dolens and the various forms of "slight cold."

*c.* The absence of nursing and its consequent reflex stimulation of the uterus.

*d.* Improper exercise before involution is complete, displacing the uterus and so interfering with its circulation and producing a passive congestion.

*e.* And, at times, insufficient exercise, with bad hygienic influences, producing deficient vital activity without at first well marked disease.

*f.* Constipation of the bowels, with the accompanying hæmorrhoids, fissures and ulcers of the anus.

*g.* Too frequent sexual intercourse; the physiological congestion of erection passing into the pathological congestion which accompanies inflammation.

Unfortunately, many of these causes may be combined in the same patient, and thus mutually aid in the certainty of the result—subinvolution.

Consequences of subinvolution.—It is perhaps the most frequent cause of the malpositions of the uterus, such as prolapsus, version and flexion. The uterus being enlarged and, consequently, heavier, its natural supports are not capable of holding it in its normal place in the pelvis, and hence the varieties of prolapsus and version, for the fœtus has gone from the uterus, and this was the normal excitant in giving tone to the nervous, vascular, and especially the muscular structure of the organs of generation. During pregnancy these organs are, when excited, very like those in a stage of erection, and from their tonic firmness, are not only as easily displaced, but after miscarriage or delivery at full term, if the uterus remains enlarged, this normal tonicity is gone and displacements easily follow.

The cavity of the uterus, too, is large and the walls, including the cervix, soft and flexible, offering favorable conditions for flexions, if the cervix is held firmly while forces from above press downward upon the fundus.

More or less engorgement with blood must necessarily accompany subinvolution; for, while pregnancy existed, all the tissues of the uterus were increased in quantity, the vascular, together with the muscular and nervous, and even the peritoneum; and this physiological congestion offers a favorable nidus for influences capable of exciting inflammation to produce their legitimate results.

The whole thickness of the uterine walls may become inflamed in this way. Metritis, including even the peritoneum and the adjacent areolar tissue; perimetritis; or the mucous membrane alone may be the location of the inflammation; endometritis and the discharges from the placental wound may produce disease of the mucous membrane around, which will extend through the cervix to the vagina, and even to the external organs; cervicitis and vaginitis and the development of the nervous tissue in the uterus renders the transmission easy of the influences that will produce inflammation.

Again, the muscular fibres are undergoing—imperfectly, it is true—a retrograde metamorphosis, offering favorable conditions for inflammation, depressed vitality with active nutrition.

The vascular structures, too, including both the lymphatic and blood vessels are in the best possible condition to absorb morbid products and to assist inflammatory processes.

A moment's attention to the size of the placental wound after delivery at full term, when the uterus measures 8, 10, or even 12 inches in depth, as compared with its size when firmly contracted, and but four or five inches in depth, will convince any one that the opportunities for septic absorption are greatly increased by imperfect contraction of the uterus, the beginning of subinvolution. Indeed, the dangers do not correspond with the size of the placental wound, but are even greater.

After miscarriage, though the placental wound is not as large and the lymphatic and blood vessels not as highly developed, the remains of placental tissue and blood clots are quite as likely to be present and induce septicæmia. And the muscular structure not being as much developed does not contract as thoroughly.

The unusual development of the nervous structures in the uterus, in subinvolution, explains the reflex symptoms of pelvic disease so constantly complained of by such patients and dated back by them, in their origin, to a miscarriage, or the birth of a child.

This excessive development of the nervous structures of the uterus, connecting with both the sympathetic and spinal systems of nerves, renders reflex transmission easy to any part of the body. Hence the gynæcologist is not surprised to find the symptoms of pelvic disease, especially in subinvolution, reflected to any part of the body.

The following illustrative cases were to me exceedingly suggestive and instructive :

*Case I.*—Mrs. C. S., married, age 36, Swede, servant, doing general housework. Gave birth to twins 13 years since. No pregnancy before or since. No headache or other symptom of uterine disease, except some backache and the dysuria for which the patient consulted me. At first, not knowing or suspecting the patient had been married or borne children, I prescribed simple diuretics and then other diuretics with the bromides. Not getting the least relief from the dysuria (there was no cystitis), and it had already continued six or eight weeks, I made more careful inquiry into the patient's previous history, and found, as stated above, that she had given birth to twins 13 years previous, but had had no special symptoms of pelvic disease, except during the past two months. Examined uterus July 25th, 1873. Very much enlarged — nearly 12½ centimeters in depth — but little other evidence of disease ; a simple case of subinvolution of 13 years standing. A single application of the fluid solution of carbolic acid to the uterus relieved the dysuria at once, all internal medication being omitted. The relief was so complete that it was difficult to prevail upon the patient to have any further local treatment. But the same application was made at the end of a week, August 9th, the same local application, with the fluid extract of ergot, 2 C. C., three times a day internally, the only internal treatment since local applications were commenced. August 16th, treatment continued; uterus 11½ Cm. The ergot was continued but a short time; for the patient could not be prevailed upon to take it—not appreciating the need of it. Five more applications were made of carbolic acid, making nine in all, at intervals of one or two weeks, and November 13th my record was: uterus, 9 Cm.; patient thinks herself well, and will not come for further treatment.

*Case II.*—Mrs. E. W. S., American, age 35, music teacher, married 7 years, one miscarriage 4 years since, not pregnant before or since, menses regular, some dysmenorrhœa; bowels loose, especially after menses, 9 or 10 stools a day, about 4 at other times; urine hot and high colored when stools are frequent, not at other times—very anæmic; uterus examined Oct. 8th, 1873. Endometritis, some anteflexion, uterus 9 Cm. The first application to the uterus stopped the diarrhœa without any other treatment, and it never returned. Twelve subsequent applications were made between this time and May 7th, 1874, when, my record says, she seems entirely cured, though still somewhat anæmic. During this time, iron, strychnine and other tonics were given internally, though none during the first six months of treatment. Saw patient in the summer of 1878; no return of diarrhœa, still moderately anæmic.

*Treatment of subinvolution.*—The treatment is readily suggested by the nature of the cause or causes in each particular case, and without specifying the kind for each class of cases, I will simply emphasize the importance of watching the uterus after delivery at full term, and especially after miscarriage, to determine whether involution is going on normally, and if not to prevent subinvolution by internal, and if need be, local treatment.

All cases of confinement, whether premature or at full term, should be under the care of a competent physician during the whole period of involution, and this is normally from one to two months, and not one to two weeks, the usual time of attendance. And by weekly examinations, he should determine whether involution is going on normally or not, and if not, he should adopt such measures as will assist it.

The use of the corn or rye ergot immediately after delivery, and during the few days following, if the uterus does not remain firmly contracted, will usually ensure such a result. And by preventing the formation of clots in the uterus, it will, in great measure prevent after pains in multipara, and as there will be no clots to decompose, this source of septicæmia will be avoided.

The use of quinine or some other anti-periodic at this same time, in doses up to, or just short of cinchonism, is likely to prevent the onset of the milder forms of fever, and by circulating in the blood, to act as an antiseptic in preventing the severer forms, and so indirectly, if not directly, facilitate involution.

I will mention but two other remedies; strychnia, and the mineral acids, as likely to aid digestion and promote the normal contraction of the uterus, by their action upon the nervous system.

If, at the end of a month, six weeks, or at the most two months, the depth of the uterus is not less than four inches, local treatment should be commenced and continued with appropriate internal remedies until involution is complete.

No parturient woman should be allowed to resume her full duties, or consider herself fully recovered, until involution is complete. If she does, she risks her future health, and perhaps even her life; and no physician fullfils his duty who does not instruct his patients in these things.

